# A new performance evaluation indicator for the LEE Jong-wook Fellowship Program of Korea Foundation for International Healthcare to better assess its long-term educational impacts: a Delphi study

**DOI:** 10.3352/jeehp.2024.21.27

**Published:** 2024-10-02

**Authors:** Minkyung Oh, Bo Young Yoon

**Affiliations:** 1 Department of Pharmacology, Inje University College of Medicine, Busan, Korea; 2 Department of Internal Medicine, Inje University College of Medicine, Busan, Korea; Hallym University, Korea

**Keywords:** Analytic hierarchy process, Delivery of health care, Fellowships and scholarships, Program evaluation, Republic of Korea

## Abstract

**Purpose:**

The Dr. LEE Jong-wook Fellowship Program, established by the Korea Foundation for International Healthcare (KOFIH), aims to strengthen healthcare capacity in partner countries. The aim of the study was to develop new performance evaluation indicators for the program to better assess long-term educational impact across various courses and professional roles.

**Methods:**

A 3-stage process was employed. First, a literature review of established evaluation models (Kirkpatrick’s 4 levels, context/input/process/product evaluation model, Organization for Economic Cooperation and Development Assistance Committee criteria) was conducted to devise evaluation criteria. Second, these criteria were validated via a 2-round Delphi survey with 18 experts in training projects from May 2021 to June 2021. Third, the relative importance of the evaluation criteria was determined using the analytic hierarchy process (AHP), calculating weights and ensuring consistency through the consistency index and consistency ratio (CR), with CR values below 0.1 indicating acceptable consistency.

**Results:**

The literature review led to a combined evaluation model, resulting in 4 evaluation areas, 20 items, and 92 indicators. The Delphi surveys confirmed the validity of these indicators, with content validity ratio values exceeding 0.444. The AHP analysis assigned weights to each indicator, and CR values below 0.1 indicated consistency. The final set of evaluation indicators was confirmed through a workshop with KOFIH and adopted as the new evaluation tool.

**Conclusion:**

The developed evaluation framework provides a comprehensive tool for assessing the long-term outcomes of the Dr. LEE Jong-wook Fellowship Program. It enhances evaluation capabilities and supports improvements in the training program’s effectiveness and international healthcare collaboration.

## Graphical abstract

[Fig f4-jeehp-21-27]

## Introduction

### Background

The Dr. LEE Jong-wook Fellowship Program is a project being implemented by the Korea Foundation for International Healthcare (KOFIH), and it is also part of the memorial project to honor the late Dr. Lee Jong-Wook, who served as secretary-general of the World Health Organization (WHO) from January 2003 to May 2006. This program is a mid- to long-term invitational training project for healthcare personnel in partner countries to improve the level of healthcare in partner countries and promote international cooperation, supported by the KOFIH. Since its launch in 2007, 1,504 healthcare practitioners from 30 countries have participated as of 2023. This program helps practitioners to learn about the healthcare technology and system of Korea while contributing in the improvement of global healthcare services and building healthcare systems.

As the program continues, the importance and internal/external needs for educational evaluation also increase. However, it is difficult to measure long-term effects with current performance indicators other than achieving short-term goals, and it is also challenging to collect, analyze, and interpret information that matches various expertise, such as that of doctors, nurses, and biomedical engineers. Additionally, current performance indicators have already reached their highest measurable levels.

Therefore, we planned to develop objective performance indicators for evaluating and monitoring educational effectiveness by course and job group by reviewing and analyzing existing performance indicators and sought to improve the program’s evaluation system by developing new indicators that can measure the effectiveness of training in the long term. Furthermore, we aimed to contribute to promoting the revision of the KOFIH’s training projects and revitalizing international exchange through a systematic evaluation system.

### Objectives

The purpose of this study was to develop a new performance evaluation indication for the LEE Jong-wook Fellowship Program through a 3-stage process: (1) literature review of established models, (2) validation using a Delphi survey, and (3) importance assessment using the analytic hierarchy process (AHP).

## Methods

### Ethics statement

We obtained consent from the 18 experts who participated in the Delphi panel.

### Study design

We used the Delphi method to evaluate the appropriateness of the assessment tool developed through a literature review. In order to develop criteria for evaluating the LEE Jong-wook Fellowship Program, we designed this study in 3 stages: devising evaluation criteria, validating the evaluation criteria, and calculating the relative importance of the evaluation criteria. First, we reviewed research related to the program and drafted program evaluation criteria. Second, we conducted 2-stage Delphi surveys on the developed evaluation criteria, targeting a panel of experts to evaluate the validity of the evaluation criteria. Third, we calculated the relative importance of the evaluation criteria using the AHP method [1-3].

The Delphi surveys were designed in 3 rounds to confirm the validity of the evaluation indicators. In the first 2 rounds, content validity ratio (CVR) values were calculated to check whether the indicators were valid. In the last of the 3 rounds, the weight for each evaluation indicator was calculated using the AHP method.

### Setting

Researchers developed criteria and evaluation indicators after negotiating a proposal based on a literature review with staff and team leaders involved in the LEE Jong-wook Fellowship Program. Then, a finalized survey paper was written for the Delphi survey.

### Participants

#### Respondent group

Delphi members consisted of a total of 18 people (Table 1). Delphi members formed a panel of academic experts related to training projects, experts in international development cooperation projects, and medical education experts who had training experience at the KOFIH in order to synthesize various expert opinions. Experts working at public institutions were also included upon recommendation by the KOFIH.

#### Workgroup

This study involved 8 researchers, 5 of whom had experience in qualitative research, 2 of whom had extensive experience in qualitative research and Delphi research, and the other had extensive experience in Official Development Assistance projects.

### Instrumentation

An email requesting participation in the Delphi panel was sent to experts to confirm their willingness to participate. This Delphi study was conducted from May 2021 to June 2021. It took approximately 30–40 minutes to respond to each survey. Then, the first, second, and third Delphi surveys were conducted on a weekly basis. The survey questions were delivered by email for each round. The collected survey questionnaires were analyzed, and the survey questionnaire was revised to reflect the results. In the third AHP survey, in cases where inconsistent results occurred, this was explained to the relevant panel member, and the survey paper was re-distributed and collected for revision and re-analysis.

### Protocol

**Fig. 1.**
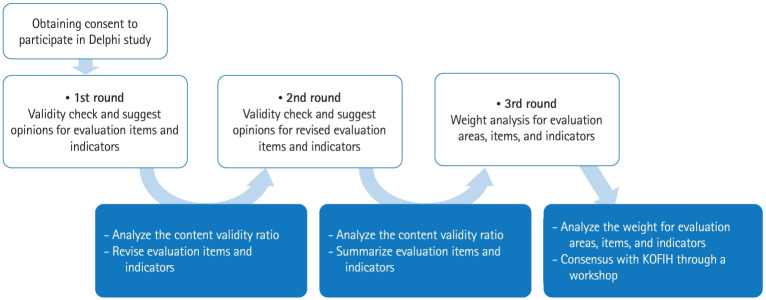
Flow chart of the study protocol. KOFIH, Korea Foundation for International Healthcare.

The study protocol is available in Fig. 1.

### Statistical methods

The data were summarized using descriptive statistics, and the CVR was calculated for the Delphi panel. The CVR is the proportional level of agreement regarding how many experts within a panel rate an item as “essential” and is calculated in the following equation [4,5]:


$$\text{CVR}=\frac{n_{e}-\left(\frac{N}{2}\right)}{\frac{N}{2}}$$

where *n_e_*=number of panel members indicating an item as “essential” and N=number of panel members. Since the number of experts in the Delphi panel was 18, validity was considered to have been confirmed if the CVR was 0.444 or higher. The weight for each evaluation indicator was calculated using the AHP. The equation for the eigenvector method to find the priority vector in AHP is as follows:


$$\text{A}\cdot \text{W}=\lambda_{max}\cdot W$$

where A is a pairwise comparison matrix for evaluation indicators; *λ_max_* is the maximum eigenvalue; and *W* is the eigenvector. The consistency index (CI) was calculated to determine how consistently respondents responded to relative importance:


$$\text{CI}=\frac{\lambda_{max}-n}{n-1}$$

The consistency ratio (CR) was calculated using the Randon index (RI) as follows:


$$\text{CR}=\frac{CI}{RI}$$

If the CR value was less than 0.1, it was judged that consistency had been ensured, and if it exceeded 0.1, a pairwise comparison was performed again or the questionnaire was revised.

## Results

**Fig. 2.**
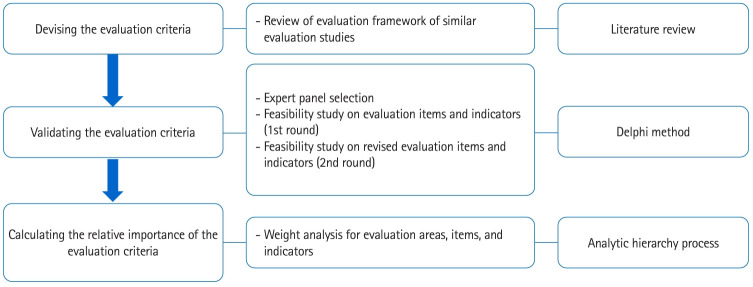
Research process and design.

This study was conducted in 3 stages, and the research content and research methods of each stage were as follows (Fig. 2).

### Devising the evaluation criteria based on the literature review

We reviewed the literature to examine several models for evaluating training programs. Kirkpatrick’s 4-level model is a famous and widely used model for evaluating outcomes in training programs and this model can be used to specifically evaluate program outcomes [6]. Level 1 is “response,” which involves evaluating the response to the training and satisfaction with the training; level 2 is “learning,” where the knowledge, skills, and attitude changes gained from the training are evaluated; level 3 is “behavior,” which involves evaluating the behavioral changes of the trainees, and level 4 is “result,” where the final outcomes of the training are evaluated. However, Kirkpatrick’s 4-level model does not include trainees’ motivation, variables of knowledge and skills, important relationships between program elements and situations, or the effectiveness of resources.

The context/input/process/product (CIPP) evaluation model focuses on improving programs. This model accommodates the ever-changing nature of educational programs, as well as educators’ needs [7]. This model can evaluate all stages of an educational program, including planning, implementation, overall process, and final results, in accordance with the flow of situations, inputs, processes, and outputs. However, to evaluate a program using the CIPP evaluation model, care must be taken from the program planning stage.

The LOGIC model approach is primarily used when evaluating the relationships between program components and program context [8]. This is a logic model consisting of inputs, activities, outputs, and outcomes. Its format is not complicated, but it may oversimplify the program and prevent evaluators from producing what they need. This model shares similar characteristics with the CIPP evaluation model, but focuses on systems that embody change processes and innovation in education.

**Fig. 3.**
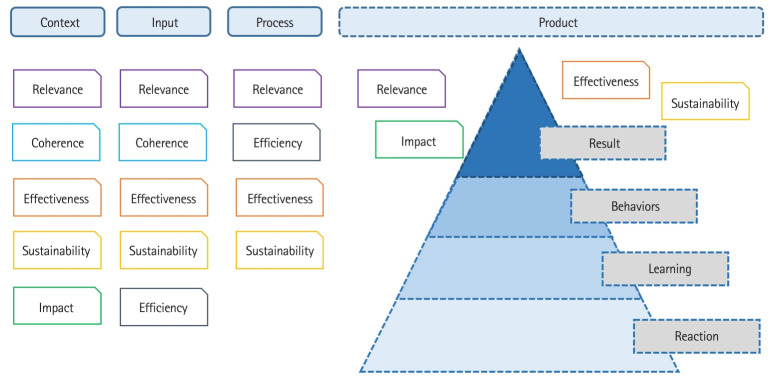
Evaluation model and evaluation criteria.

Based on the literature review of reports, papers and expert advice [9], CIPP and Kirkpatrick’s 4-level model were evaluated as appropriate. In addition to these models, evaluation criteria were selected, including 6 evaluation criteria (consistency, sustainability, efficiency, relevance, impact, and effectiveness) recommended by the Organization for Economic Cooperation and Development (OECD) Development Assistance Committee (DAC) [10,11]. Based on this evaluation model, evaluation criteria were derived by dividing them into input, activity, and output performance (Fig. 3).

### Validating the evaluation criteria

We developed a draft of evaluation criteria and evaluation indicators and conducted validity verification through a Delphi survey targeting a panel of experts. We sent an email requesting participation in the Delphi panel to all experts to confirm their willingness to participate, and then conducted the first, second, and third Delphi surveys on a weekly basis (Datasets 1–3). The collected questionnaires for each round were analyzed, and the next questionnaire was revised based on these. A workshop was conducted to complete the analysis of the Delphi results. It was established that the CVR for each evaluation index must be at least 0.444 to ensure validity. The first round Delphi survey consisted of 4 evaluation areas, 20 evaluation items, and 85 evaluation indicators. The evaluation criteria were well organized and the indicators in each area were evaluated as valid by the Delphi panel members. Comments were provided on items that required detailed evaluation or had ambiguous aspects of measurement. The second round of the survey was conducted by revising the indicators based on the quantitative and qualitative opinions of the Delphi panel. The second Delphi survey consisted of 4 evaluation areas, 20 evaluation items, and 92 evaluation indicators. The CVR of the items and indicators across all areas was 0.444 or higher, indicating that content validity was secured (Table 2).

### Calculating the relative importance of the evaluation criteria

We surveyed experts on the relative importance of the finalized evaluation criteria. In this study, a CR analysis was conducted on all response values for domains, items, and indicators based on the results of the expert panel’s responses, and it was determined that consistency was maintained if the value of the CR did not exceed 0.1 (Table 3). The final weights of the areas, items, and indicators were calculated by taking the arithmetic mean of the individual weights of the panel, where consistency was ensured (Table 4). The final evaluation indicators were confirmed through a workshop with the KOFIH and selected as the evaluation tool (Table 5).

## Discussion

### Key results

The study developed a new set of performance evaluation indicators for the Dr. LEE Jong-wook Fellowship Program, addressing the need for objective and long-term measures of educational effectiveness. Through a comprehensive 3-stage process—reviewing the literature, validating with a Delphi panel of 18 experts, and using the AHP to determine the relative importance of the indicators—the researchers integrated elements from Kirkpatrick’s 4 levels, the CIPP model, and OECD DAC criteria.

### Interpretation

#### Devising evaluation criteria

The study developed evaluation criteria by reviewing established models such as Kirkpatrick’s 4-level framework, the CIPP model, and the OECD DAC criteria. This combination ensured a holistic evaluation approach, addressing both short-term outcomes like training satisfaction and long-term aspects such as sustainability and impact.

#### Validation through the Delphi study

The Delphi survey involved 18 experts who reviewed and validated the proposed evaluation indicators over 2 rounds. In the second round, 92 indicators were confirmed, achieving a minimum CVR of 0.444, reflecting the result of consensus among experts. This validation process confirmed that the indicators were suitable and reliable for evaluating the fellowship program’s diverse training objectives and outcomes.

#### Calculating importance using AHP

The importance of each evaluation criterion was ranked using the AHP. The consistency of expert feedback was ensured, and the CR was less than 0.1, indicating that the developed evaluation index was reliable.

This innovative hybrid model ensures a robust, nuanced framework that strengthens the program’s evaluation system. The evaluation model used in this study can ensure a robust and detailed framework for improving the evaluation system of educational programs.

### Comparison with previous studies

Compared to previous studies that typically use only a single model [12,13], this study attempted to provide a more comprehensive and robust evaluation framework by integrating multiple models (Kirkpatrick’s 4-level model, the CIPP model, and the OECD DAC criteria). In addition, by using both the Delphi method and the AHP to validate and prioritize evaluation indicators, it provides a level of rigor that has often been lacking in previous studies that have relied more on subjective or less formal methods. Unlike past studies that primarily evaluated immediate outcomes such as satisfaction and learning [14], this study provides a more holistic approach to evaluating the effectiveness of educational programs by emphasizing long-term impacts such as behavior change and sustainability.

### Limitations

This study’s specific focus on the Dr. LEE Jong-wook Fellowship Program means that while the methodology can be applied to other contexts, the criteria and indicators developed may require adjustments for applicability in different contexts or programs. Furthermore, the inherent difficulty in measuring long-term impacts remains a challenge, as the study primarily focused on immediate and intermediate outcomes, which may not fully capture the long-term effectiveness and sustainability of the training programs.

### Suggestions

The performance indicators developed in this study are educational program evaluation indicators. In particular, since they are used to evaluate the performance of a training program, it is more meaningful to discuss the cycle of evaluating performance indicators and how to use them while establishing an overall program evaluation plan. For further study, it would be beneficial to apply the developed evaluation framework to a broader range of healthcare training programs across different countries and contexts to test its adaptability and generalizability. Additionally, conducting a longitudinal study to track the long-term impacts of the training programs on participants’ professional development and healthcare outcomes in their home countries would provide deeper insights into the sustained effectiveness and impact of the training.

### Conclusion

We effectively developed a comprehensive set of performance evaluation indicators for the Dr. LEE Jong-wook Fellowship Program, addressing the need for objective and long-term assessment measures. By integrating multiple evaluation models and validating them through a Delphi panel of experts, the study produced a robust framework that enhances the program’s evaluation system and contributes significantly to global healthcare capacity building. Despite limitations related to the panel size and the challenge of measuring long-term impacts, these indicators can serve as a valuable tool for improving healthcare training programs and lay the groundwork for future research to further refine and test them across diverse contexts.

## Figures and Tables

**Figure f4-jeehp-21-27:**
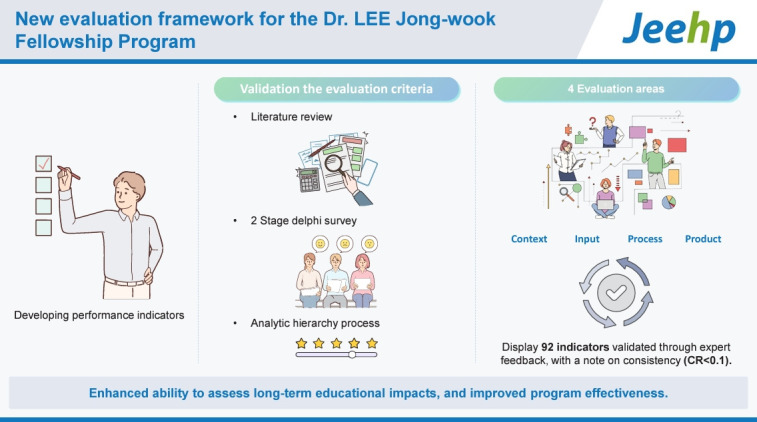


**Table 1.** List of Delphi panel experts

ID	Affiliation	Name	Field of expert	Position
1	A university	JOO	TP	Professor
2		KOO	ODA, ME	Professor
3		LOO	TP	Professor
4		MOO	TP	Professor
5		NOO	ME	Professor
6		OOO	ODA, ME	Professor
7	B university	POO	TP	Professor
8		QOO	TP	Team leader
9		ROO	TP	Professor
10		SOO	TP	Professor
11	C university	TOO	ODA	Team leader
12	D hospital	UOO	TP	Team leader
13	E university	VOO	ODA	Professor
14	F association	WOO	ODA	Chairman
15		XOO	TP	PhD
16	G university	YOO	ODA, ME	Professor
17	H institute	ZOO	ODA	Professor
18	I center	AOO	TP	Professor

TP, academic experts related to training projects; ODA, experts in international development cooperation projects; ME, medical education experts.

**Table 2.** Validity of the evaluation criteria

Evaluation areas	CVR		Evaluation items	CVR		Evaluation indicators	CVR	
	1st	2nd		1st	2nd		1st	2nd
Context	1.00	1.00	Needs assessment and program goal setting	1.00	1.00	Appropriateness of demand surveys	0.89	0.89
			Linkages between projects	0.67	0.89	Linkage rate of training projects with other internal and external ODA projects	0.44	0.67
			Project sustainability	0.78	1.00	Feasibility and integration rate of mid-to-long-term proposals after project completion	0.67	0.89
Input	0.89	0.89	Training outcomes and curriculum	0.89	0.89	Curriculum that aligns with the training outcome	1.00	1.00
			Input of material and human resources	0.89	0.89	Adequacy of material resources	1.00	1.00
						Adequacy of human resources	1.00	1.00
			Investment to strengthen KOFIH’s competence	0.89	0.78	Performance of the internal capacity of KOFIH strengthening program	0.44	0.78
Process	1.00	1.00	Training preparation	0.67	0.78	Adherence to the training preparation process	0.78	0.89
			Training implementation and evaluation	0.78	1.00	Feedback and assessment for trainee	1.00	1.00
			Post-training management	0.67	1.00	Conducting a post-training management program that meets its intended outcomes	0.78	1.00
			Monitoring	0.78	1.00	Compliance with monitoring guidelines	0.56	0.78
Product	1.00	1.00	Response	1.00	1.00	Training satisfaction	1.00	1.00
			Learning	0.89	1.00	Competency achievement	0.89	1.00
			Behavior	0.78	1.00	Continuity of trainees’ roles	0.78	1.00
						Job performance improvement/application of trained performance	0.89	1.00
						Continuity of exchange	0.44	0.78
			Result	0.67	0.78	Achievement	0.78	0.89
						Influence	0.67	0.78
						Diffusion	0.67	0.89

CVR, content validity ratio; ODA, experts in international development cooperation projects; KOFIH, Korea Foundation for International Healthcare.

**Table 3.** Consistency of the evaluation criteria

Evaluation areas	CI/CR	Consistency	Evaluation items	CI/CR	Consistency	Evaluation indicators	CI/CR	Consistency
Context	0.03/0.03	Y	Needs assessment and program goal setting	0.04/0.04	Y	Appropriateness of demand surveys	0.05/0.06	Y
			Linkages between projects			Linkage rate of training projects with other internal and external ODA projects	0.00/0.00	-
			Project sustainability			Feasibility and integration rate of mid-to-long-term proposals after project completion	0.00/0.00	-
Input			Training outcomes and curriculum	0.05/0.09	Y	Curriculum that aligns with the training outcome	0.06/0.07	Y
			Input of material and human resources			Adequacy of material resources	0.10/0.07	Y
						Adequacy of human resources	0.10/0.07	Y
			Investment to strengthen KOFIH’s competence			Performance of the internal capacity of KOFIH strengthening program	0.03/0.06	Y
Process			Training preparation	0.03/0.04	Y	Adherence to the training preparation process	0.00/0.00	-
			Training implementation and evaluation			Feedback and assessment for trainee	0.02/0.05	Y
			Post-training management			Conducting a post-training management program that meets its intended outcomes	0.00/0.00	Y
			Monitoring			Compliance with monitoring guidelines	0.03/0.03	Y
Product			Response	0.12/0.09	Y	Training satisfaction	0.10/0.09	Y
			Learning			Competency achievement	0.07/0.05	Y
			Behavior			Continuity of trainees’ roles	0.00/0.00	Y
						Job performance improvement/application of trained performance	0.00/0.00	Y
						Continuity of exchange	0.00/0.00	N
			Result			Achievement	0.00/0.00	Y
						Influence	0.00/0.00	Y
						Diffusion	0.04/0.07	Y

CI, consistency index; CR, consistency ratio; ODA, experts in international development cooperation projects; KOFIH, Korea Foundation for International Healthcare.

**Table 4.** Relative importance of the evaluation criteria

Evaluation areas	Weight	Evaluation items	Weight	Evaluation indicators	Weight
Context	0.16	Needs assessment and program goal setting	0.31	Appropriateness of demand surveys	0.39
		Linkages between projects	0.11	Linkage rate of training projects with other internal and external ODA projects	0.53
		Project sustainability	0.21	Feasibility and integration rate of mid-to-long-term proposals after project completion	0.41
Input	0.19	Training outcomes and curriculum	0.45	Curriculum that aligns with the training outcome	0.37
		Input of material and human resources	0.41	Adequacy of material resources	0.40
				Adequacy of human resources	0.28
		Investment to strengthen KOFIH’s competence	0.14	Performance of the internal capacity of KOFIH strengthening program	0.25
Process	0.32	Training preparation	0.23	Adherence to the training preparation process	0.46
		Training implementation and evaluation	0.44	Feedback and assessment for trainee	0.41
		Post-training management	0.19	Conducting a post-training management program that meets its intended outcomes	0.64
		Monitoring	0.13	Compliance with monitoring guidelines	0.28
Product	0.33	Response	0.15	Training satisfaction	0.15
		Learning	0.23	Competency achievement	0.23
		Behavior	0.10	Continuity of trainees’ roles	0.10
				Job performance improvement/application of trained performance	0.16
				Continuity of exchange	0.04
		Result	0.31	Achievement	0.16
				Influence	0.07
				Diffusion	0.08

ODA, experts in international development cooperation projects; KOFIH, Korea Foundation for International Healthcare.

**Table 5.** The final evaluation indicators

Evaluation areas	OECD DAC	Evaluation items	Evaluation indicators
Context	Relevance	Needs assessment and program goal setting	Appropriateness of demand surveys
	Coherence	Linkages between projects	Linkage rate of training projects with other internal and external ODA projects
	Sustainability	Project sustainability	Feasibility and integration rate of mid-to-long-term proposals after project completion
Input	Relevance	Training outcomes and curriculum	Curriculum that aligns with the training outcomes
	Effectiveness/efficiency	Input of material and human resources	Adequacy of material resources
			Adequacy of human resources
		Investment to strengthen KOFIH’s competence	Performance of the internal capacity of KOFIH strengthening program
Process	Effectiveness/sustainability	Training preparation	Adherence to the training preparation process
	Effectiveness	Training implementation and evaluation	Feedback and assessment for trainee
	Effectiveness/Sustainability	Post-training management	Conducting a post-training management program that meets its intended outcomes
	Effectiveness	Monitoring	Compliance with monitoring guidelines
Product	Relevance	Response	Training satisfaction
	Effectiveness	Learning	Competency achievement
	Relevance/effectiveness/sustainability	Behavior	Continuity of trainees’ roles
			Job performance improvement/application of trained performance
			Continuity of exchange
	Relevance/impact/sustainability	Result	Achievement
			Influence
			Diffusion

OECD DAC, Organization for Economic Cooperation and Development Development Assistance Committee; ODA, experts in international development cooperation projects; KOFIH, Korea Foundation for International Healthcare.

## Footnotes

**Data availability**
Data files are available from Harvard Dataverse: https://doi.org/10.7910/DVN/RTPIZB Dataset 1. Raw response data at 1st round Delphi survey.

Dataset 3. Raw response data at 3rd Analytic Hierarchy Process survey.

## Supplementary materials

Supplement 1. Audio recording of the abstract.
